# *cis*-Bis{1,1-dibenzyl-3-[(furan-2-yl)carbon­yl]thio­ureato-κ^2^ *O*,*S*}nickel(II)

**DOI:** 10.1107/S1600536811010749

**Published:** 2011-03-26

**Authors:** Hiram Pérez, Cecilia C. P. da Silva, Ana M. Plutín, Carlos A. de Simone, Javier Ellena

**Affiliations:** a Departamento de Química Inorgánica, Facultad de Química, Universidad de la Habana, Habana 10400, Cuba; b Grupo de Cristalogafia, Instituto de Física de São Carlos, Universidade de São Paulo, São Carlos, Brazil; c Laboratorio de Síntesis Orgánica, Facultad de Química, Universidad de la Habana, Habana 10400, Cuba

## Abstract

In the title compound, [Ni(C_20_H_17_N_2_O_2_S)_2_], the Ni^II^ atom is coordinated by the S and O atoms of two 1,1-dibenzyl-3-[(furan-2-yl)carbon­yl]thio­ureate ligands in a distorted square-planar geometry. The two O and two S atoms are mutually *cis* to each other. The Ni—S and Ni—O bond lengths lie within the range of those found in related structures. The dihedral angle between the planes of the two chelating rings is 20.33 (6)°.

## Related literature

For general background to transition metal complexes with *N*-acyl disubstituted thio­ureas, see: Arslan *et al.* (2003 ▶). For details of the synthesis, see: Nagasawa & Mitsunobu (1981 ▶). For related structures, see: Binzet *et al.* (2009 ▶); Ozer *et al.* (2009 ▶); Pérez *et al.* (2009 ▶).
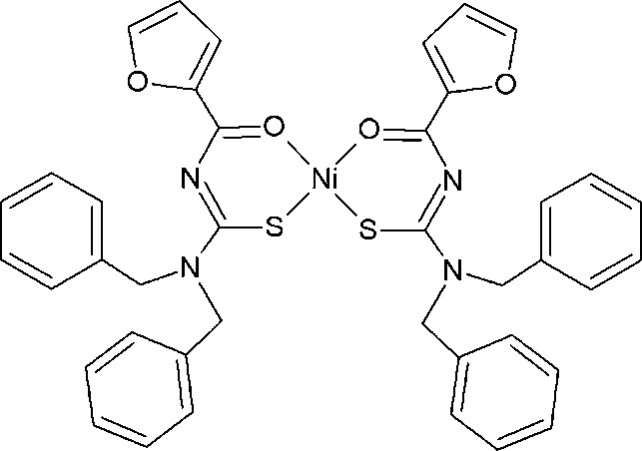


## Experimental

### 

#### Crystal data

[Ni(C_20_H_17_N_2_O_2_S)_2_]
*M* *_r_* = 757.54
Monoclinic, 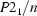

*a* = 18.7260 (4) Å
*b* = 10.8430 (2) Å
*c* = 19.6490 (5) Å
β = 114.628 (1)°
*V* = 3626.72 (14) Å^3^
*Z* = 4
Mo *K*α radiation
μ = 0.70 mm^−1^
*T* = 293 K
0.38 × 0.27 × 0.19 mm

#### Data collection

Nonius KappaCCD diffractometer
Absorption correction: Gaussian (Coppens *et al.*, 1965 ▶) *T*_min_ = 0.779, *T*_max_ = 0.886
28443 measured reflections
7636 independent reflections
6155 reflections with *I* > 2σ(*I*)
*R*_int_ = 0.058

#### Refinement

*R*[*F* ^2^ > 2σ(*F* ^2^)] = 0.039
*wR*(*F* ^2^) = 0.106
*S* = 1.09
7636 reflections
460 parameters
H-atom parameters constrained
Δρ_max_ = 0.30 e Å^−3^
Δρ_min_ = −0.55 e Å^−3^

### 

Data collection: *COLLECT* (Enraf–Nonius, 2000 ▶); cell refinement: *SCALEPACK* (Otwinowski & Minor, 1997 ▶); data reduction: *DENZO* (Otwinowski & Minor, 1997 ▶) and *SCALEPACK*; program(s) used to solve structure: *SHELXS97* (Sheldrick, 2008 ▶); program(s) used to refine structure: *SHELXL97* (Sheldrick, 2008 ▶); molecular graphics: *ORTEP-3 for Windows* (Farrugia, 1997 ▶) and *Mercury* (Macrae *et al.*, 2006 ▶); software used to prepare material for publication: *WinGX* (Farrugia, 1999 ▶).

## Supplementary Material

Crystal structure: contains datablocks global, I. DOI: 10.1107/S1600536811010749/bt5498sup1.cif

Structure factors: contains datablocks I. DOI: 10.1107/S1600536811010749/bt5498Isup2.hkl

Additional supplementary materials:  crystallographic information; 3D view; checkCIF report

## Figures and Tables

**Table 1:** Selected bond lengths (Å)

Ni—O2	1.8645 (14)
Ni—O1	1.8664 (13)
Ni—S1	2.1392 (6)
Ni—S2	2.1444 (5)

**Fig. 1.**
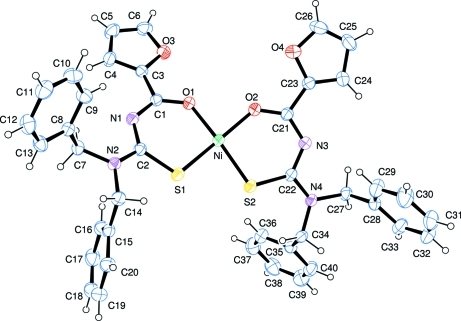
The molecular structure of title compound. Displacement ellipsoids are drawn at the 30% probability level.

**Fig. 2.**
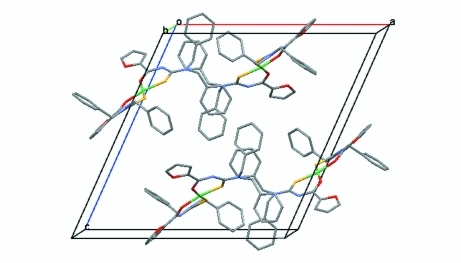
Crystal packing of title complex, viewed along [010]. H atoms have been omitted for clarity.

## supplementary crystallographic information

### Comment

*N*-Acyl-*N*',*N*'-disubstituted thioureas are well known as chelating agents. Over recent years, many transition metal complexes with *N*-acyl disubstituted thioureas have been reported (Arslan *et al.*, 2003). During complex formation, the ligand is deprotonated, which results in a neutral complex with a six-membered ring chelating metal ion.

In the crystal structure of the title complex, the two furoylthiourea molecules adopt a *cis* conformation, bonded to the central Ni^II^ ion as shown in Fig. 1. The complex coordination geometry is  distorted square-planar as reflected by angles O1–Ni–S2 [169.66 (5)°] and O2–Ni–S1 [170.09 (6)°]. The distance of nickel atom from the best plane through the coordination sphere is 0.0255 Å. The chelate ring systems, Ni–O1–C1–N2–C2—S1 and Ni–O2–C21–N3–C22–S2, are nearly planar with the largest deviations from the best plane being -0.149 (1)Å for S1 and -0.130 (2)Å for C22, respectively. The dihedral angle of 20.33 (6)° between these chelate planes indicates a strong distortion from square planarity towards tetrahedral geometry. By comparison, the corresponding O–N–S angles and dihedral angle for the diphenyl analog (Pérez *et al.*, 2009) are 176.26 (8)°, 176.87 (8)° and 5.74 (2)°, respectively, As result, the square-planar coordination geometry of the title molecule is significantly more distorted. The Ni–S and Ni–O bond lengths lie within the range of those found in the related structures (Binzet *et al.*, 2009, Ozer *et al.*, 2009). The bond lengths of the thiocarbonyl and carbonyl bonds are longer than the average for C=S and C=O, while the C–N bonds in the chelate rings are all shorter than the average for C–N single bond of about 1.48 Å. This indicate extensive electronic delocalization within the complex rings. Fig. 2 shows the arrangement of the complex molecules in the unit cell.

### Experimental

The 1,1-dibenzyl-3-[(furan-2-yl)carbonyl]thiourea ligand was prepared using the standard procedure previously reported in the literature (Nagasawa & Mitsunobu, 1981) by the reaction of furoyl chloride with KSCN in anhydrous acetone, and then condensation with dibenzylamine. To an ethanol solution (30 ml) containing the ligand (0.70 g, 2 mmol) was added an ethanol solution of Ni(CH_3_COO)_2_.4H_2_O (0.25 g, 1 mmol). The solution was stirred at room temperature for 2 h, and at once a solution of NaOH (1 N) was added to adjust pH to the neutral value. The mixture was filtered and the filtrate was evaporated under reduced pressure to give a red solid, which was washed with acetone. Single crystals were obtained by slow evaporation of a chloroform/dichloromethane solution (1:1, *v*/*v*) of the complex.

### Refinement

H atoms were positioned geometrically and refined as riding atoms, with C—H = 0.93 Å and *U*_iso_(H) = 1.2*U*_eq_(C) for aromatic, and C—H = 0.97 Å and 1.5*U*_eq_(C) for methylene H atoms.

### Crystal data

**Table:**

[Ni(C_20_H_17_N_2_O_2_S)_2_]	*F*(000) = 1576
*M**_r_* = 757.54	*D*_x_ = 1.387 Mg m^−^^3^
Monoclinic, *P*2_1_/*n*	Mo *K*α radiation, λ = 0.71073 Å
Hall symbol: -P 2yn	Cell parameters from 16796 reflections
*a* = 18.7260 (4) Å	θ = 2.9–27.1°
*b* = 10.8430 (2) Å	µ = 0.70 mm^−^^1^
*c* = 19.6490 (5) Å	*T* = 293 K
β = 114.628 (1)°	Prism, blue
*V* = 3626.72 (14) Å^3^	0.38 × 0.27 × 0.19 mm
*Z* = 4	

### Data collection

**Table:**

Nonius KappaCCD diffractometer	7636 independent reflections
Radiation source: Enraf Nonius FR590	6155 reflections with *I* > 2σ(*I*)
graphite	*R*_int_ = 0.058
Detector resolution: 9 pixels mm^-1^	θ_max_ = 27.1°, θ_min_ = 3.0°
CCD rotation images, thick slices scans	*h* = −23→23
Absorption correction: gaussian (Coppens *et al.*, 1965)	*k* = −13→11
*T*_min_ = 0.779, *T*_max_ = 0.886	*l* = −22→24
28443 measured reflections	

### Refinement

**Table:**

Refinement on *F*^2^	Primary atom site location: structure-invariant direct methods
Least-squares matrix: full	Secondary atom site location: difference Fourier map
*R*[*F*^2^ > 2σ(*F*^2^)] = 0.039	Hydrogen site location: inferred from neighbouring sites
*wR*(*F*^2^) = 0.106	H-atom parameters constrained
*S* = 1.09	*w* = 1/[σ^2^(*F*_o_^2^) + (0.0504*P*)^2^ + 0.7766*P*] where *P* = (*F*_o_^2^ + 2*F*_c_^2^)/3
7636 reflections	(Δ/σ)_max_ = 0.001
460 parameters	Δρ_max_ = 0.30 e Å^−^^3^
0 restraints	Δρ_min_ = −0.55 e Å^−^^3^

### Special details

**Table:**

Geometry. All s.u.'s (except the s.u. in the dihedral angle between two l.s. planes) are estimated using the full covariance matrix. The cell s.u.'s are taken into account individually in the estimation of s.u.'s in distances, angles and torsion angles; correlations between s.u.'s in cell parameters are only used when they are defined by crystal symmetry. An approximate (isotropic) treatment of cell s.u.'s is used for estimating s.u.'s involving l.s. planes.
Refinement. Refinement of *F*^2^ against ALL reflections. The weighted *R*-factor *wR* and goodness of fit *S* are based on *F*^2^, conventional *R*-factors *R* are based on *F*, with *F* set to zero for negative *F*^2^. The threshold expression of *F*^2^ > 2σ(*F*^2^) is used only for calculating *R*-factors(gt) *etc*. and is not relevant to the choice of reflections for refinement. *R*-factors based on *F*^2^ are statistically about twice as large as those based on *F*, and *R*- factors based on ALL data will be even larger.

### Fractional atomic coordinates and isotropic or equivalent isotropic displacement parameters (Å^2^)

**Table:**

	*x*	*y*	*z*	*U*_iso_*/*U*_eq_	
Ni	0.491462 (13)	0.08249 (2)	0.203939 (13)	0.04117 (9)	
S1	0.45918 (4)	0.27092 (5)	0.17502 (4)	0.05827 (16)	
S2	0.40661 (3)	0.08767 (5)	0.25086 (3)	0.05258 (14)	
O1	0.54999 (8)	0.06931 (12)	0.14719 (8)	0.0473 (3)	
O2	0.53333 (8)	−0.07065 (14)	0.24394 (9)	0.0540 (4)	
O3	0.60150 (9)	−0.03183 (13)	0.05316 (9)	0.0580 (4)	
O4	0.64021 (9)	−0.24664 (17)	0.30736 (10)	0.0699 (5)	
N1	0.55377 (9)	0.26690 (14)	0.09920 (9)	0.0430 (3)	
N2	0.52359 (9)	0.45372 (15)	0.13379 (9)	0.0419 (3)	
N3	0.45963 (9)	−0.14303 (15)	0.30784 (9)	0.0451 (4)	
N4	0.34078 (9)	−0.08247 (14)	0.30160 (9)	0.0427 (4)	
C1	0.56213 (10)	0.14477 (17)	0.10394 (10)	0.0405 (4)	
C2	0.51798 (10)	0.33035 (17)	0.13450 (10)	0.0407 (4)	
C3	0.59370 (11)	0.09342 (17)	0.05366 (11)	0.0421 (4)	
C4	0.61673 (14)	0.1442 (2)	0.00348 (13)	0.0581 (5)	
H4	0.6167	0.2277	−0.0075	0.07*	
C5	0.64091 (15)	0.0458 (2)	−0.02934 (14)	0.0653 (6)	
H5	0.6604	0.0521	−0.0657	0.078*	
C6	0.63044 (15)	−0.0570 (2)	0.00174 (14)	0.0623 (6)	
H6	0.6414	−0.1358	−0.01	0.075*	
C7	0.56076 (11)	0.51742 (18)	0.09058 (10)	0.0428 (4)	
H7A	0.5256	0.5819	0.061	0.051*	
H7B	0.5668	0.4589	0.056	0.051*	
C8	0.63963 (11)	0.57404 (17)	0.13665 (10)	0.0415 (4)	
C9	0.70500 (12)	0.5013 (2)	0.17242 (12)	0.0543 (5)	
H9	0.6996	0.416	0.1713	0.065*	
C10	0.77852 (13)	0.5534 (2)	0.21001 (14)	0.0639 (6)	
H10	0.8223	0.5034	0.234	0.077*	
C11	0.78655 (13)	0.6796 (3)	0.21171 (13)	0.0650 (6)	
H11	0.836	0.715	0.2364	0.078*	
C12	0.72184 (14)	0.7536 (2)	0.17704 (14)	0.0637 (6)	
H12	0.7273	0.8389	0.1787	0.076*	
C13	0.64831 (12)	0.70070 (19)	0.13954 (12)	0.0529 (5)	
H13	0.6045	0.7508	0.1162	0.063*	
C14	0.48762 (12)	0.53554 (18)	0.17003 (11)	0.0475 (4)	
H14A	0.519	0.6099	0.1858	0.057*	
H14B	0.4893	0.4951	0.2147	0.057*	
C15	0.40373 (12)	0.57173 (17)	0.12178 (12)	0.0460 (4)	
C16	0.35972 (12)	0.5213 (2)	0.05261 (12)	0.0538 (5)	
H16	0.3815	0.4608	0.0335	0.065*	
C17	0.28272 (15)	0.5602 (3)	0.01087 (16)	0.0717 (7)	
H17	0.2536	0.5266	−0.0362	0.086*	
C18	0.24990 (16)	0.6483 (3)	0.0393 (2)	0.0835 (9)	
H18	0.1984	0.674	0.0117	0.1*	
C19	0.29335 (18)	0.6984 (3)	0.1087 (2)	0.0778 (8)	
H19	0.2712	0.7578	0.1281	0.093*	
C20	0.36927 (15)	0.6610 (2)	0.14927 (15)	0.0622 (6)	
H20	0.3983	0.6958	0.196	0.075*	
C21	0.51974 (11)	−0.14077 (18)	0.28856 (10)	0.0427 (4)	
C22	0.40345 (11)	−0.05635 (17)	0.28756 (10)	0.0413 (4)	
C23	0.57549 (11)	−0.2412 (2)	0.32215 (11)	0.0484 (5)	
C24	0.57399 (15)	−0.3381 (2)	0.36413 (13)	0.0627 (6)	
H24	0.5358	−0.3555	0.3814	0.075*	
C25	0.64210 (18)	−0.4084 (3)	0.37688 (16)	0.0794 (8)	
H25	0.6575	−0.481	0.4043	0.095*	
C26	0.67957 (16)	−0.3504 (3)	0.34200 (17)	0.0831 (8)	
H26	0.7265	−0.377	0.3413	0.1*	
C27	0.33271 (12)	−0.20135 (18)	0.33465 (11)	0.0472 (4)	
H27A	0.3679	−0.2612	0.3283	0.057*	
H27B	0.2794	−0.2315	0.308	0.057*	
C28	0.35100 (12)	−0.19020 (18)	0.41685 (11)	0.0480 (4)	
C29	0.42288 (15)	−0.1481 (3)	0.46681 (14)	0.0724 (7)	
H29	0.4602	−0.1243	0.4497	0.087*	
C30	0.44037 (19)	−0.1406 (4)	0.54257 (16)	0.0917 (9)	
H30	0.489	−0.1107	0.5758	0.11*	
C31	0.3866 (2)	−0.1768 (3)	0.56872 (15)	0.0846 (8)	
H31	0.3987	−0.1723	0.6196	0.101*	
C32	0.31511 (19)	−0.2196 (3)	0.52005 (16)	0.0800 (8)	
H32	0.2785	−0.2444	0.5378	0.096*	
C33	0.29656 (15)	−0.2262 (2)	0.44355 (14)	0.0649 (6)	
H33	0.2475	−0.255	0.4105	0.078*	
C34	0.27511 (11)	0.00250 (18)	0.28685 (11)	0.0458 (4)	
H34A	0.2919	0.0857	0.2827	0.055*	
H34B	0.2607	0.0006	0.3289	0.055*	
C35	0.20404 (11)	−0.02923 (18)	0.21620 (11)	0.0450 (4)	
C36	0.20390 (14)	−0.0134 (2)	0.14626 (13)	0.0603 (6)	
H36	0.2484	0.017	0.1423	0.072*	
C37	0.13797 (17)	−0.0426 (3)	0.08225 (15)	0.0767 (7)	
H37	0.1384	−0.0317	0.0355	0.092*	
C38	0.07181 (17)	−0.0875 (3)	0.08736 (17)	0.0780 (8)	
H38	0.0275	−0.107	0.0442	0.094*	
C39	0.07148 (15)	−0.1035 (3)	0.15642 (17)	0.0774 (7)	
H39	0.0269	−0.1343	0.1601	0.093*	
C40	0.13681 (14)	−0.0741 (2)	0.22024 (15)	0.0626 (6)	
H40	0.1357	−0.0846	0.2668	0.075*	

### Atomic displacement parameters (Å^2^)

**Table:**

	*U*^11^	*U*^22^	*U*^33^	*U*^12^	*U*^13^	*U*^23^
Ni	0.03816 (14)	0.04489 (15)	0.04528 (16)	−0.00023 (9)	0.02215 (11)	0.00586 (10)
S1	0.0690 (3)	0.0462 (3)	0.0836 (4)	0.0072 (2)	0.0556 (3)	0.0105 (3)
S2	0.0562 (3)	0.0446 (3)	0.0736 (4)	0.0032 (2)	0.0436 (3)	0.0100 (2)
O1	0.0487 (7)	0.0472 (7)	0.0543 (8)	0.0060 (6)	0.0297 (7)	0.0107 (6)
O2	0.0510 (8)	0.0584 (9)	0.0628 (9)	0.0103 (6)	0.0338 (7)	0.0196 (7)
O3	0.0706 (10)	0.0444 (8)	0.0732 (10)	0.0086 (7)	0.0439 (8)	0.0062 (7)
O4	0.0584 (9)	0.0785 (11)	0.0826 (12)	0.0178 (8)	0.0391 (9)	0.0175 (9)
N1	0.0437 (8)	0.0431 (8)	0.0473 (9)	−0.0015 (7)	0.0240 (7)	0.0010 (7)
N2	0.0427 (8)	0.0418 (8)	0.0434 (8)	−0.0037 (6)	0.0202 (7)	−0.0024 (6)
N3	0.0449 (8)	0.0464 (9)	0.0481 (9)	0.0007 (7)	0.0233 (7)	0.0034 (7)
N4	0.0454 (8)	0.0411 (8)	0.0485 (9)	−0.0013 (6)	0.0263 (7)	0.0037 (7)
C1	0.0336 (8)	0.0457 (10)	0.0423 (10)	−0.0013 (7)	0.0160 (8)	0.0032 (8)
C2	0.0388 (9)	0.0436 (10)	0.0394 (9)	−0.0013 (7)	0.0160 (8)	0.0015 (8)
C3	0.0395 (9)	0.0404 (10)	0.0487 (11)	0.0000 (7)	0.0206 (8)	0.0019 (8)
C4	0.0750 (14)	0.0520 (12)	0.0632 (14)	−0.0024 (11)	0.0446 (12)	0.0032 (10)
C5	0.0773 (16)	0.0704 (15)	0.0661 (15)	0.0000 (12)	0.0478 (13)	−0.0058 (12)
C6	0.0677 (14)	0.0601 (14)	0.0692 (15)	0.0090 (11)	0.0384 (12)	−0.0090 (11)
C7	0.0430 (9)	0.0452 (10)	0.0378 (9)	−0.0056 (8)	0.0145 (8)	0.0024 (8)
C8	0.0408 (9)	0.0450 (10)	0.0390 (10)	−0.0036 (8)	0.0169 (8)	0.0004 (7)
C9	0.0483 (11)	0.0488 (11)	0.0607 (13)	0.0000 (9)	0.0177 (10)	−0.0017 (10)
C10	0.0408 (11)	0.0762 (16)	0.0655 (15)	0.0023 (10)	0.0132 (10)	−0.0016 (12)
C11	0.0485 (12)	0.0831 (17)	0.0576 (14)	−0.0205 (12)	0.0164 (11)	−0.0061 (12)
C12	0.0657 (14)	0.0516 (12)	0.0645 (14)	−0.0187 (11)	0.0177 (12)	−0.0018 (11)
C13	0.0513 (11)	0.0451 (11)	0.0558 (12)	−0.0041 (9)	0.0159 (10)	0.0033 (9)
C14	0.0545 (11)	0.0432 (10)	0.0479 (11)	−0.0028 (9)	0.0243 (9)	−0.0073 (8)
C15	0.0522 (11)	0.0392 (10)	0.0542 (12)	−0.0031 (8)	0.0296 (9)	0.0026 (8)
C16	0.0519 (11)	0.0547 (12)	0.0569 (13)	−0.0033 (9)	0.0245 (10)	0.0041 (10)
C17	0.0568 (13)	0.0806 (17)	0.0692 (16)	−0.0053 (12)	0.0180 (12)	0.0189 (13)
C18	0.0587 (14)	0.086 (2)	0.113 (2)	0.0181 (14)	0.0426 (17)	0.0433 (18)
C19	0.0799 (17)	0.0636 (15)	0.112 (2)	0.0207 (14)	0.0622 (18)	0.0204 (16)
C20	0.0729 (15)	0.0502 (12)	0.0766 (15)	0.0029 (11)	0.0440 (13)	−0.0004 (11)
C21	0.0415 (9)	0.0467 (10)	0.0406 (10)	−0.0008 (8)	0.0180 (8)	0.0007 (8)
C22	0.0442 (9)	0.0437 (10)	0.0416 (10)	−0.0039 (8)	0.0235 (8)	−0.0010 (8)
C23	0.0456 (10)	0.0560 (12)	0.0460 (11)	0.0051 (9)	0.0214 (9)	0.0032 (9)
C24	0.0737 (15)	0.0620 (14)	0.0599 (14)	0.0151 (11)	0.0353 (12)	0.0176 (11)
C25	0.095 (2)	0.0741 (17)	0.0690 (17)	0.0361 (15)	0.0340 (16)	0.0267 (13)
C26	0.0686 (16)	0.094 (2)	0.0869 (19)	0.0386 (15)	0.0327 (15)	0.0181 (16)
C27	0.0519 (11)	0.0422 (10)	0.0548 (12)	−0.0049 (8)	0.0296 (9)	0.0026 (8)
C28	0.0536 (11)	0.0435 (10)	0.0526 (11)	0.0001 (9)	0.0276 (9)	0.0072 (9)
C29	0.0638 (14)	0.096 (2)	0.0567 (14)	−0.0132 (14)	0.0248 (12)	0.0092 (13)
C30	0.0836 (19)	0.122 (3)	0.0550 (16)	−0.0111 (18)	0.0142 (14)	0.0108 (16)
C31	0.106 (2)	0.100 (2)	0.0527 (15)	0.0116 (18)	0.0377 (16)	0.0133 (14)
C32	0.097 (2)	0.094 (2)	0.0723 (18)	0.0048 (16)	0.0578 (17)	0.0112 (15)
C33	0.0686 (14)	0.0697 (15)	0.0710 (15)	−0.0074 (12)	0.0438 (13)	−0.0002 (12)
C34	0.0508 (10)	0.0452 (10)	0.0535 (11)	0.0010 (8)	0.0337 (9)	0.0007 (9)
C35	0.0479 (10)	0.0400 (10)	0.0540 (11)	0.0047 (8)	0.0281 (9)	0.0027 (8)
C36	0.0641 (13)	0.0669 (14)	0.0586 (13)	0.0011 (11)	0.0343 (11)	0.0053 (11)
C37	0.0829 (18)	0.0895 (19)	0.0533 (14)	0.0033 (15)	0.0240 (13)	0.0070 (13)
C38	0.0657 (16)	0.0734 (17)	0.0733 (18)	−0.0039 (13)	0.0077 (14)	0.0046 (13)
C39	0.0535 (13)	0.0853 (19)	0.088 (2)	−0.0106 (12)	0.0240 (14)	0.0065 (15)
C40	0.0557 (13)	0.0707 (15)	0.0687 (15)	−0.0070 (11)	0.0332 (12)	0.0066 (12)

### Geometric parameters (Å, °)

**Table:**

Ni—O2	1.8645 (14)	C15—C16	1.375 (3)
Ni—O1	1.8664 (13)	C15—C20	1.391 (3)
Ni—S1	2.1392 (6)	C16—C17	1.394 (3)
Ni—S2	2.1444 (5)	C16—H16	0.93
S1—C2	1.7296 (18)	C17—C18	1.374 (4)
S2—C22	1.7314 (19)	C17—H17	0.93
O1—C1	1.267 (2)	C18—C19	1.376 (5)
O2—C21	1.264 (2)	C18—H18	0.93
O3—C6	1.359 (3)	C19—C20	1.370 (4)
O3—C3	1.366 (2)	C19—H19	0.93
O4—C23	1.360 (2)	C20—H20	0.93
O4—C26	1.361 (3)	C21—C23	1.462 (3)
N1—C1	1.332 (2)	C23—C24	1.343 (3)
N1—C2	1.338 (2)	C24—C25	1.416 (3)
N2—C2	1.342 (2)	C24—H24	0.93
N2—C14	1.465 (2)	C25—C26	1.326 (4)
N2—C7	1.475 (2)	C25—H25	0.93
N3—C21	1.329 (2)	C26—H26	0.93
N3—C22	1.342 (2)	C27—C28	1.510 (3)
N4—C22	1.343 (2)	C27—H27A	0.97
N4—C34	1.466 (2)	C27—H27B	0.97
N4—C27	1.479 (2)	C28—C29	1.372 (3)
C1—C3	1.457 (3)	C28—C33	1.383 (3)
C3—C4	1.348 (3)	C29—C30	1.387 (4)
C4—C5	1.415 (3)	C29—H29	0.93
C4—H4	0.93	C30—C31	1.366 (4)
C5—C6	1.325 (3)	C30—H30	0.93
C5—H5	0.93	C31—C32	1.361 (4)
C6—H6	0.93	C31—H31	0.93
C7—C8	1.504 (2)	C32—C33	1.397 (4)
C7—H7A	0.97	C32—H32	0.93
C7—H7B	0.97	C33—H33	0.93
C8—C9	1.377 (3)	C34—C35	1.509 (3)
C8—C13	1.381 (3)	C34—H34A	0.97
C9—C10	1.383 (3)	C34—H34B	0.97
C9—H9	0.93	C35—C40	1.383 (3)
C10—C11	1.376 (3)	C35—C36	1.384 (3)
C10—H10	0.93	C36—C37	1.383 (4)
C11—C12	1.374 (3)	C36—H36	0.93
C11—H11	0.93	C37—C38	1.373 (4)
C12—C13	1.386 (3)	C37—H37	0.93
C12—H12	0.93	C38—C39	1.370 (4)
C13—H13	0.93	C38—H38	0.93
C14—C15	1.510 (3)	C39—C40	1.376 (4)
C14—H14A	0.97	C39—H39	0.93
C14—H14B	0.97	C40—H40	0.93
			
O2—Ni—O1	86.28 (6)	C16—C17—H17	120
O2—Ni—S1	170.14 (6)	C19—C18—C17	119.8 (2)
O1—Ni—S1	94.92 (4)	C19—C18—H18	120.1
O2—Ni—S2	95.67 (4)	C17—C18—H18	120.1
O1—Ni—S2	169.62 (5)	C20—C19—C18	120.1 (3)
S1—Ni—S2	84.91 (2)	C20—C19—H19	120
C2—S1—Ni	108.33 (7)	C18—C19—H19	120
C22—S2—Ni	108.40 (6)	C19—C20—C15	121.2 (3)
C1—O1—Ni	131.73 (12)	C19—C20—H20	119.4
C21—O2—Ni	131.10 (13)	C15—C20—H20	119.4
C6—O3—C3	106.39 (16)	O2—C21—N3	130.40 (18)
C23—O4—C26	105.89 (19)	O2—C21—C23	116.69 (16)
C1—N1—C2	122.99 (16)	N3—C21—C23	112.81 (17)
C2—N2—C14	122.98 (15)	N3—C22—N4	115.81 (16)
C2—N2—C7	122.04 (15)	N3—C22—S2	126.65 (14)
C14—N2—C7	114.77 (16)	N4—C22—S2	117.41 (14)
C21—N3—C22	123.70 (16)	C24—C23—O4	110.23 (19)
C22—N4—C34	124.04 (15)	C24—C23—C21	131.78 (19)
C22—N4—C27	122.13 (16)	O4—C23—C21	117.90 (17)
C34—N4—C27	113.82 (15)	C23—C24—C25	106.4 (2)
O1—C1—N1	129.82 (17)	C23—C24—H24	126.8
O1—C1—C3	116.43 (17)	C25—C24—H24	126.8
N1—C1—C3	113.68 (16)	C26—C25—C24	106.6 (2)
N1—C2—N2	116.65 (16)	C26—C25—H25	126.7
N1—C2—S1	126.82 (15)	C24—C25—H25	126.7
N2—C2—S1	116.34 (13)	C25—C26—O4	110.9 (2)
C4—C3—O3	109.47 (17)	C25—C26—H26	124.5
C4—C3—C1	133.14 (19)	O4—C26—H26	124.5
O3—C3—C1	117.37 (16)	N4—C27—C28	112.38 (16)
C3—C4—C5	106.6 (2)	N4—C27—H27A	109.1
C3—C4—H4	126.7	C28—C27—H27A	109.1
C5—C4—H4	126.7	N4—C27—H27B	109.1
C6—C5—C4	106.7 (2)	C28—C27—H27B	109.1
C6—C5—H5	126.6	H27A—C27—H27B	107.9
C4—C5—H5	126.6	C29—C28—C33	118.8 (2)
C5—C6—O3	110.8 (2)	C29—C28—C27	120.63 (19)
C5—C6—H6	124.6	C33—C28—C27	120.6 (2)
O3—C6—H6	124.6	C28—C29—C30	120.7 (2)
N2—C7—C8	115.16 (15)	C28—C29—H29	119.7
N2—C7—H7A	108.5	C30—C29—H29	119.7
C8—C7—H7A	108.5	C31—C30—C29	120.4 (3)
N2—C7—H7B	108.5	C31—C30—H30	119.8
C8—C7—H7B	108.5	C29—C30—H30	119.8
H7A—C7—H7B	107.5	C32—C31—C30	119.8 (3)
C9—C8—C13	118.95 (18)	C32—C31—H31	120.1
C9—C8—C7	120.94 (17)	C30—C31—H31	120.1
C13—C8—C7	119.98 (17)	C31—C32—C33	120.3 (3)
C10—C9—C8	120.9 (2)	C31—C32—H32	119.8
C10—C9—H9	119.5	C33—C32—H32	119.8
C8—C9—H9	119.5	C28—C33—C32	120.1 (3)
C9—C10—C11	119.6 (2)	C28—C33—H33	119.9
C9—C10—H10	120.2	C32—C33—H33	119.9
C11—C10—H10	120.2	N4—C34—C35	112.60 (16)
C12—C11—C10	120.2 (2)	N4—C34—H34A	109.1
C12—C11—H11	119.9	C35—C34—H34A	109.1
C10—C11—H11	119.9	N4—C34—H34B	109.1
C11—C12—C13	119.9 (2)	C35—C34—H34B	109.1
C11—C12—H12	120.1	H34A—C34—H34B	107.8
C13—C12—H12	120.1	C40—C35—C36	118.4 (2)
C8—C13—C12	120.4 (2)	C40—C35—C34	120.16 (19)
C8—C13—H13	119.8	C36—C35—C34	121.42 (18)
C12—C13—H13	119.8	C37—C36—C35	120.4 (2)
N2—C14—C15	114.88 (16)	C37—C36—H36	119.8
N2—C14—H14A	108.5	C35—C36—H36	119.8
C15—C14—H14A	108.5	C38—C37—C36	120.4 (3)
N2—C14—H14B	108.5	C38—C37—H37	119.8
C15—C14—H14B	108.5	C36—C37—H37	119.8
H14A—C14—H14B	107.5	C39—C38—C37	119.6 (3)
C16—C15—C20	118.4 (2)	C39—C38—H38	120.2
C16—C15—C14	123.75 (18)	C37—C38—H38	120.2
C20—C15—C14	117.8 (2)	C38—C39—C40	120.3 (2)
C15—C16—C17	120.5 (2)	C38—C39—H39	119.9
C15—C16—H16	119.7	C40—C39—H39	119.9
C17—C16—H16	119.7	C39—C40—C35	120.9 (2)
C18—C17—C16	120.0 (3)	C39—C40—H40	119.5
C18—C17—H17	120	C35—C40—H40	119.5
			
O1—Ni—S1—C2	−13.85 (8)	C16—C17—C18—C19	0.4 (4)
S2—Ni—S1—C2	176.58 (7)	C17—C18—C19—C20	0.2 (4)
O2—Ni—S2—C22	−10.43 (9)	C18—C19—C20—C15	−0.4 (4)
O1—Ni—S2—C22	90.0 (3)	C16—C15—C20—C19	−0.1 (3)
S1—Ni—S2—C22	179.46 (7)	C14—C15—C20—C19	−179.9 (2)
O2—Ni—O1—C1	−173.40 (18)	Ni—O2—C21—N3	17.7 (3)
S1—Ni—O1—C1	−3.21 (17)	Ni—O2—C21—C23	−166.36 (15)
S2—Ni—O1—C1	85.4 (3)	C22—N3—C21—O2	−8.0 (3)
O1—Ni—O2—C21	−174.92 (19)	C22—N3—C21—C23	175.98 (18)
S2—Ni—O2—C21	−5.16 (19)	C21—N3—C22—N4	169.86 (18)
Ni—O1—C1—N1	19.1 (3)	C21—N3—C22—S2	−14.4 (3)
Ni—O1—C1—C3	−164.31 (13)	C34—N4—C22—N3	177.04 (17)
C2—N1—C1—O1	−10.8 (3)	C27—N4—C22—N3	−2.3 (3)
C2—N1—C1—C3	172.54 (16)	C34—N4—C22—S2	0.8 (3)
C1—N1—C2—N2	170.64 (17)	C27—N4—C22—S2	−178.49 (14)
C1—N1—C2—S1	−14.5 (3)	Ni—S2—C22—N3	21.39 (19)
C14—N2—C2—N1	−179.94 (16)	Ni—S2—C22—N4	−162.87 (13)
C7—N2—C2—N1	5.6 (3)	C26—O4—C23—C24	−0.3 (3)
C14—N2—C2—S1	4.7 (2)	C26—O4—C23—C21	−177.2 (2)
C7—N2—C2—S1	−169.83 (13)	O2—C21—C23—C24	−171.6 (2)
Ni—S1—C2—N1	24.97 (18)	N3—C21—C23—C24	5.1 (3)
Ni—S1—C2—N2	−160.18 (12)	O2—C21—C23—O4	4.6 (3)
C6—O3—C3—C4	0.4 (2)	N3—C21—C23—O4	−178.80 (18)
C6—O3—C3—C1	179.04 (18)	O4—C23—C24—C25	0.3 (3)
O1—C1—C3—C4	−176.7 (2)	C21—C23—C24—C25	176.7 (2)
N1—C1—C3—C4	0.5 (3)	C23—C24—C25—C26	−0.2 (3)
O1—C1—C3—O3	5.1 (3)	C24—C25—C26—O4	0.0 (4)
N1—C1—C3—O3	−177.75 (17)	C23—O4—C26—C25	0.2 (3)
O3—C3—C4—C5	−0.7 (3)	C22—N4—C27—C28	102.7 (2)
C1—C3—C4—C5	−179.0 (2)	C34—N4—C27—C28	−76.7 (2)
C3—C4—C5—C6	0.7 (3)	N4—C27—C28—C29	−58.2 (3)
C4—C5—C6—O3	−0.4 (3)	N4—C27—C28—C33	124.0 (2)
C3—O3—C6—C5	0.0 (3)	C33—C28—C29—C30	−0.7 (4)
C2—N2—C7—C8	−107.9 (2)	C27—C28—C29—C30	−178.5 (3)
C14—N2—C7—C8	77.1 (2)	C28—C29—C30—C31	1.0 (5)
N2—C7—C8—C9	71.4 (2)	C29—C30—C31—C32	−0.6 (5)
N2—C7—C8—C13	−112.9 (2)	C30—C31—C32—C33	−0.1 (5)
C13—C8—C9—C10	−0.8 (3)	C29—C28—C33—C32	0.0 (4)
C7—C8—C9—C10	175.0 (2)	C27—C28—C33—C32	177.8 (2)
C8—C9—C10—C11	0.0 (4)	C31—C32—C33—C28	0.4 (4)
C9—C10—C11—C12	0.8 (4)	C22—N4—C34—C35	101.7 (2)
C10—C11—C12—C13	−0.7 (4)	C27—N4—C34—C35	−78.9 (2)
C9—C8—C13—C12	0.8 (3)	N4—C34—C35—C40	110.8 (2)
C7—C8—C13—C12	−175.0 (2)	N4—C34—C35—C36	−69.8 (2)
C11—C12—C13—C8	−0.1 (4)	C40—C35—C36—C37	−0.3 (3)
C2—N2—C14—C15	−87.8 (2)	C34—C35—C36—C37	−179.7 (2)
C7—N2—C14—C15	87.0 (2)	C35—C36—C37—C38	0.1 (4)
N2—C14—C15—C16	8.0 (3)	C36—C37—C38—C39	−0.1 (4)
N2—C14—C15—C20	−172.23 (18)	C37—C38—C39—C40	0.3 (4)
C20—C15—C16—C17	0.8 (3)	C38—C39—C40—C35	−0.6 (4)
C14—C15—C16—C17	−179.4 (2)	C36—C35—C40—C39	0.6 (4)
C15—C16—C17—C18	−1.0 (4)	C34—C35—C40—C39	180.0 (2)
